# Improving ITC studies of cyclodextrin inclusion compounds by global analysis of conventional and non-conventional experiments

**DOI:** 10.3762/bjoc.10.275

**Published:** 2014-11-11

**Authors:** Eléonore Bertaut, David Landy

**Affiliations:** 1 Univ Lille Nord de France, F-59000 Lille, France; 2 ULCO, UCEIV, F-59140 Dunkerque, France

**Keywords:** cyclodextrins, global analysis, inclusion compounds, isothermal titration calorimetry, non-conventional experiments

## Abstract

The study of 1:1 cyclodextrin inclusion compounds by isothermal titration calorimetry was explored in a theoretical and experimental point of view to compare the efficiency of conventional and non-conventional experiments. All direct and competitive protocols were described and evaluated in terms of accuracy on both binding constant and inclusion enthalpy. Significant improvement in the calorimetric characterization may be obtained by means of the global analysis of non-conventional experiments coupled to the standard titration protocol. While the titration-release approach proved to be the most accurate strategy for classical complexations, the valuable contribution of other non-conventional experiments was demonstrated for issues concerning weak stability, enthalpy, or solubility.

## Introduction

The stability and thermodynamics of cyclodextrin inclusion compounds in solutions may be investigated with various analytical techniques ranging from spectroscopy to chromatography and including calorimetrical tools [1–4]. Within this scope, isothermal titration calorimetry (ITC) is gaining more and more importance, as it allows a complete thermodynamic description of the inclusion phenomena (binding constant *K*, inclusion enthalpy Δ*H*, inclusion entropy Δ*S*, heat capacity variation Δ*C_p_* ) [5–11].

On a methodological point of view, most ITC studies are based on the classical titration protocol. While one partner of the studied phenomenon, called titrand, is placed inside the cell, the second partner, called titrant, is contained in the syringe of the ITC device. Each injection of one partner on the other induces the formation of supramolecules, thus releasing an amount of heat which is proportional to the binding enthalpy and to the amount of complexes formed during this addition. The binding isotherm is then obtained by recording heats for all injections. At this stage, a fitting procedure between experimental and theoretical data allows the evaluation of various thermodynamic parameters. Alternatively, a competitive experiment may also be undertaken to investigate the titrant–titrand supramolecule, even if it is more scarcely used [12–13]. In this protocol, the titrant injections are applied to a solution containing both the titrand and a competitive molecule. Such an experiment allows the characterization of the titrant–titrand complex, provided that a preliminary titration between the titrand and the competitive molecule (this time in the syringe) has been realized and analyzed. As the data treatments of these competitive and non-competitive experiments are integrated in commercial ITC software, these two methods constitute the conventional ITC protocols.

In addition to these standard experiments, other combinations of partners between cell and syringe may be investigated, and thus some non-conventional protocols have emerged during the last decade [14–17]. Nonetheless, as the corresponding dedicated treatments are not implemented within commercial ITC software, their use remains limited. As a consequence, most of these methods have not been applied to cyclodextrin complexes. Moreover, not all the non-conventional experiments have been described to date. Finally, while titration efficiency has been thoroughly studied [7,18–19], few is known about the efficiency of non-conventional ITC protocols.

This work aims for the description of the whole conventional and non-conventional ITC experiments within the cyclodextrin context and the evaluation of the resulting accuracy on thermodynamic parameters of inclusion compounds. For this purpose, we successfully applied a unified treatment to competitive and non-competitive experiments for 1:1 Ibuprofen (IBU) complexes which are formed with β-cyclodextrin (β-CD) and hydroxypropyl-β-cyclodextrin (HPβ-CD) covering temperature ranges from 283 K to 313 K. These experimental studies were used as starting points for theoretical investigations on the efficiency of all protocols. We used covariance matrixes to calculate the uncertainty in binding constant and inclusion enthalpy, not only for the individual analysis of each experiment, but also for the global analysis of coupled experiments. All these investigations were aimed at designing the experimental strategies which achieve the highest quality in the characterization of host–guest supramolecules.

## Results and Discussion

### Non-competitive experiments

In the first part of this work, we consider the study of inclusion compounds in non-competitive conditions, i.e., with one host and one guest. The design of ITC experiments may be seen as a combination of the studied partners in both cell and syringe. For non-competitive protocols, each compartment may contain none, one or two of the studied species. Among the sixteen possible combinations, the four experiments which involve the same solution in both compartments provide no information on the complexation phenomenon. Four other combinations, involving the buffer solution and a solution containing only one partner, constitute the blank experiments to be subtracted to the real experiments. As a consequence, eight experiments may be designed to study the complexation phenomenon.

Concerning the host, guest, and host–guest solutions to be prepared, some theoretical and experimental facts have to be considered. Firstly, as cyclodextrins are often used to solubilize hydrophobic guests, titration experiments generally employ the cyclodextrin solution in the syringe and the guest solution in the cell. However, care has to be taken of the cyclodextrin concentration, since highly concentrated solutions may induce self-aggregation [20] and a high heat of dilution. In the case of β-CD, we have experimentally observed that heats of dilution were negligible for concentrations of up to 5 mM. Secondly, the ratio between host and guest concentrations in their respective stock solutions should be at least equal to ten in order to obtain a satisfying sample of the binding isotherm during the titration experiment. This implies that the guest solution should not exceed a 0.5 mM concentration (for a titrant concentration equal to 5 mM); this concentration may even be weaker, in agreement with the guest solubility. At last, the total concentration of the guest could be raised within the host and guest mixture (used for non-conventional experiments) as a consequence of the inclusion compound formation. If *S*_G_ corresponds to the solubility of the guest when prepared alone, the guest concentration ([G]_T_) which can be solubilized by a CD solution ([CD]_T_) is related to the complex stability (*K*_CD-G_) by Equation 1 [21]:

[1]
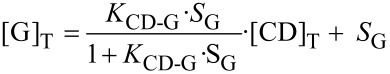


**Figure 1:**
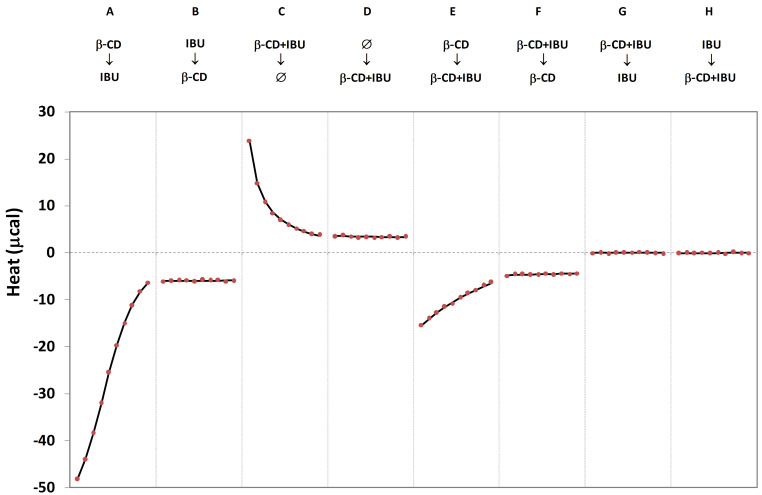
Non-competitive ITC isotherms for the β-CD-IBU complex at 298 K. Each protocol (from A to H) is defined by the respective content of syringe (top) and cell (bottom). Ø indicates the buffer solution. Lines and dots correspond to theoretical and experimental data, respectively. The x-axis corresponds to the injection number (10 injections per isotherm).

All these rules have been observed for each real and hypothetical system studied in this work. In order to explore the eight achievable ITC protocols, we choose to study the β-CD-IBU system at ambient conditions (298 K). Three solutions were prepared: (i) 5 mM β-CD solution, (ii) 0.5 mM IBU solution and (iii) 5 mM β-CD + 4.6 mM IBU solution (inducing a free IBU concentration equal to 0.5 mM). The experiments resulting from the combinations of these three solutions in cell and syringe are presented in Figure 1.

As one can see, a global analysis of the eight experiments leads to a strong agreement between experimental and theoretical data, on the basis of the treatment described in the experimental part. Such a quality in the fitting results demonstrates that a unique formalism may describe all non-competitive ITC experiments. A formation constant of 9558 M^−1^ and an inclusion enthalpy of −3326 cal·mol^−1^ have been obtained by this global approach, which lead to high accuracy (±129 M^−1^ and ±13 cal·mol^−1^ for *K*_β-CD-IBU_ and Δ*H*_β-CD-IBU_, respectively) owing to the high amount of experimental information. The stability is in agreement with the range of affinity (7000–14000 M^−1^) found in most published studies [22–27], even if some extreme values have also been observed [28–29]. The specificities of each individual experiment are described in the following.

Protocol A corresponds to the classical titration experiment. The injection of β-CD solution on IBU solution leads to the formation of inclusion compounds, and thus to the release of heat. As the concentration of free IBU molecules in the cell are decreasing after each injection, heat is diminishing all along the titration, since less and less complexes are formed. Protocol B (called “reverse titration”) corresponds to the reverse of protocol A, but in this case the host is systematically in excess if compared to the injected guest: as a result, the heat release is rather weak and constant, inducing a flat isotherm.

Protocol C implies the injection of the β-CD-IBU mixture into the buffer solution. As each injection induces a dilution of the syringe content within the cell, the complex initially formed in the mixture partially dissociates in the cell, resulting in heat consumption and positive peaks. As the inclusion compound concentration is increasing in the cell, the dissociation is diminishing injection after injection. This experiment is known as the release protocol; it has been introduced by Heerklotz [14] for vesicles and then applied to cyclodextrins [30–31]. Protocol D (called “dilution”) corresponds to the reverse of protocol C: buffer injections within the host–guest mixture induce weak dilutions, thus dissociating a weak part of inclusion compounds. As a result, rather low and constant positive peaks are observed.

Protocol E (called “attenuated titration”) and F (called “attenuated reverse titration”) corresponds to the mixing of β-CD-IBU mixture with β-CD solution. For both experiments, negative peaks are produced since the concentration of inclusion compounds increases upon injections. These experiments are similar to protocols A and B, since free IBU concentrations are equal in all experiments. Nevertheless, attenuated heats are observed as a consequence of the presence of cyclodextrin in both compartments.

Protocol G (called “annihilated release”) and H (called “annihilated dilution”) correspond to the mixing of β-CD-IBU mixture with IBU solution. Independent from the particular solutions in the cell and syringe, no heat is observed: no formation or dissociation takes place during these experiments, since the free IBU concentration is the same in both solutions owing to the experimental total concentrations used in this study. It has to be mentioned that if total concentrations do not strictly respect the equivalence of the free IBU concentrations in both solutions, positive or negative heats may be observed.

As these eight protocols lead to dissimilar isotherms, they do not involve the same accuracy on thermodynamic parameters. To evaluate the differences between protocols, we estimated, by the use of covariance matrixes, the uncertainty on binding constants and inclusion enthalpies resulting from individual analysis. These uncertainties are expressed as the standard deviations (δ*K* and δΔ*H*, respectively) multiplied by the student *t* factor (1.96). According to low values of Wiseman criteria for some of the studied complexes, total concentrations must be considered as perfectly known for the estimation of uncertainty [32]. In order to cover the range of stability generally observed in cyclodextrin chemistry, our simulations have been realized for four formation constants ranging from 10^2^ M^−1^ to 10^5^ M^−1^ (for a guest solubility equal to 2 mM). Results are presented in Table 1.

**Table 1:** Theoretical uncertainties of *K* and Δ*H* for the eight non-competitive ITC protocols with a guest solubility equal to 2 mM.

*K*	100 M^−1^		1000 M^−1^		10000 M^−1^		100000 M^−1^	
Protocol	*t*·δ*K*	*t*·δΔ*H*	*t*·δ*K*	*t*·δΔ*H*	*t*·δ*K*	*t*·δΔ*H*	*t*·δ*K*	*t*·δΔ*H*

A	>100%	>100%	11%	6%	5%	1%	5%	1%
B	>100%	75%	12%	7%	>100%	4%	>100%	4%
C	35%	19%	6%	1%	19%	7%	45%	26%
D	>100%	48%	>100%	61%	>100%	>100%	>100%	>100%
E	>100%	>100%	38%	12%	8%	1%	9%	1%
F	>100%	>100%	43%	2%	>100%	66%	>100%	>100%
G	>100%	>100%	>100%	>100%	>100%	>100%	>100%	>100%
H	>100%	>100%	>100%	>100%	>100%	>100%	>100%	>100%

**Figure 2:**
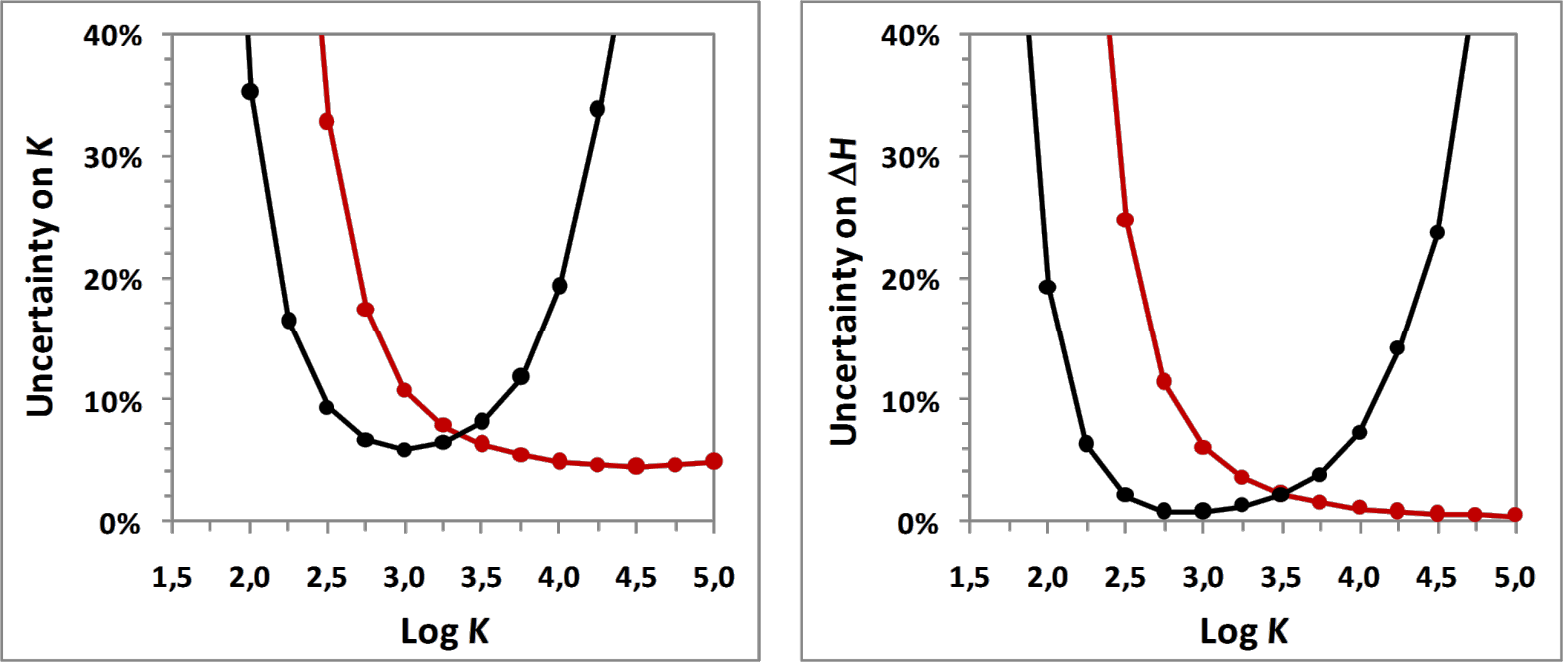
Theoretical uncertainty on *K* (left) and Δ*H* (right) as a function of log *K* for titration (red curve) and release (black curve) protocols given an aqueous guest solubility equal to 2 mM and for an inclusion enthalpy equal to −3000 cal·mol^−1^.

Numerous experiments lead to uncertainties superior to the measured variables, as a consequence of rather weak and/or flat isotherms, which are not well suited to individual analysis. At the opposite, titration and release experiments (protocols A and C, respectively) provide the most satisfying results, considering that attenuated titration (protocol E) is rather efficient but always less accurate than titration. Therefore, deeper investigations were undertaken for a better understanding of the performances of the release protocol, in comparison to the classical titration procedure. Thus, the uncertainty resulting from both titration and release procedures were compared for various guest solubilities (0.1, 0.5, 2 and 5 mM) and for a wide range of binding constant (1.5 < Log *K* < 5, i.e., 31 < *K* < 10^5^ M^−1^). Results for a guest solubility equal to 2 mM are illustrated in Figure 2 (for other solubilities, see Supporting Information File 1).

As one can see, titration uncertainty is decreasing with increasing affinity, which is in agreement with the enhancement of the Wiseman criterion [5]. As only very high formation constants may distort accuracy, no rising of uncertainty is observed for cyclodextrin complexes, explaining the overall shape depicted in Figure 2. In the case of release experiments, the uncertainty also begins by diminishing when stability is increasing. Indeed, very weak binding constants imply strong dissociation upon dilution but also weak concentrations of complex initially present in the syringe. Thus, few complexes are destroyed, resulting in low peaks and thus in relatively weak accuracy. As the stability increases, more and more complexes are formed in the syringe, allowing the dissociation of a higher amount of complex upon dilution and inducing stronger peaks. If this phenomenon results in a weaker uncertainty for moderate values of *K*, the increase of binding constants also implies weaker dissociation upon dilution: above a given value of affinity, the increase of the host–guest amount in the syringe does not compensate the drop of the inclusion dissociation in the cell in such a way that the heat signals decrease and the uncertainty grows. This explains the quadratic form of the uncertainty as a function of the stability.

When we compare the two protocols, it becomes clear that release experiments are more suited to weak affinities, while titration procedures are more efficient for stronger complexes. The release protocol must be preferred to titration experiments for binding constants inferior to approximately 3000–4000 M^−1^. This range is very slightly depending on the guest solubility, and it may be considered as a general threshold. As a result, a wide range of cyclodextrin complexes would benefit from the release protocol. Unfortunately, this is not the case in the literature, probably due to the lack of the corresponding data treatment in commercial ITC software.

**Figure 3:**
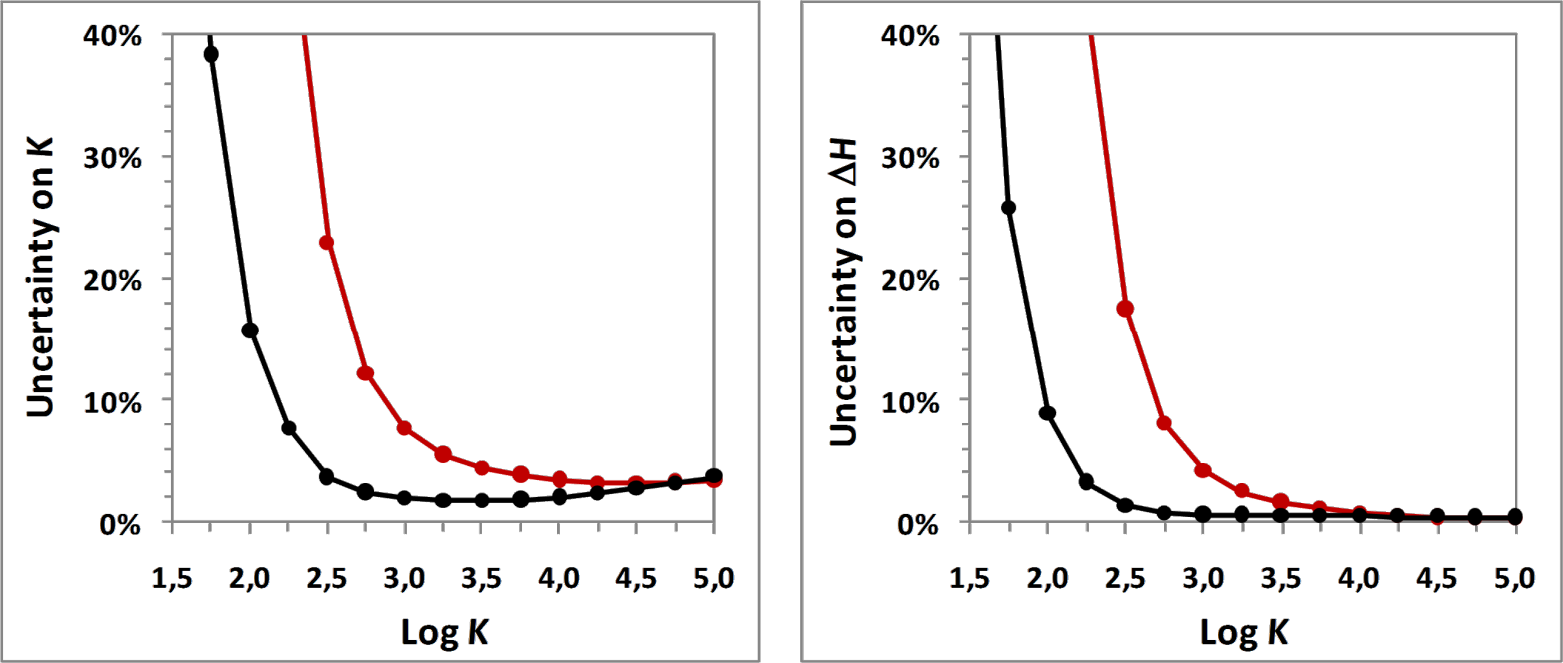
Theoretical uncertainty on *K* (left) and Δ*H* (right) as a function of Log *K* for titration-titration (red curve) and titration-release (black curve) approaches given an aqueous guest solubility equal to 2 mM and for an inclusion enthalpy equal to −3000 cal·mol^−1^.

Since titration and release protocols differ from each other, one can think that complexation studies could be improved by a global analysis of both experiments. If we employ one set of thermodynamic parameters to simultaneously fit the two thermograms, data treatment could benefit from the information contained in both isotherms. We thus evaluated the uncertainty resulting from a global analysis of titration and release protocols for various guest solubilities and binding constants, in comparison to the global analysis of two equivalent titration experiments. Results for 2 mM guest solubility are illustrated in Figure 3 (for other solubilities, see Supporting Information File 1).

As one can see, the coupling of titration and release is systematically more accurate than the analysis of two titration experiments (or at least as accurate). The improvement by the titration-release approach is especially high for weak to moderate binding constants. For instance, if we consider a binding constant of 1000 M^−1^ (log *K* = 3), the accuracy of the titration-release approach is superior to titrations by a factor close to 4 in terms of binding constant, and by a factor close to 7 for inclusion enthalpy. In addition to be beneficial for the wide majority of cyclodextrin complexes, the titration-release approach could also extend the applicability of ITC, since a high uncertainty obtained by titration in the case of problematic complexes might be reduced by this coupling strategy.

Moreover, these results demonstrate the efficiency of the global analysis concept, which may also be applied to other non-conventional experiments. For instance, it may be interesting to observe the accuracy resulting from the global analysis of protocols B and G, which are non-informative when analyzed individually (see Table 1). Against all expectations, such coupling becomes more efficient than the standard titration protocol for binding constant inferior to 4000 M^−1^, and even than the titration-release approach for binding constant inferior to 400 M^−1^ (for 2 mM guest solubility; see Supporting Information File 1). Indeed, the absence of heat for the annihilated release (protocol G) comes from the equivalence of free guest concentrations in both solutions, which is strongly depending on the formation constant. At the opposite, the constant heat which is observed for the reverse titration (protocol B) is weakly depending on the stability constant, but strongly linked to the inclusion enthalpy. As a result, the coupling of protocols B and G aggregates accurate information on both stability and enthalpy, leading to satisfying accuracy, with the assumption of well-established total concentrations. However, care should be taken with such coupling in the case of significant uncertainty on molecular quantities. In brief, while the titration-release approach constitutes the best compromise between accuracy and robustness over a wide range of binding constants, the coupling of reverse titration and annihilated release may be of great help for guests exhibiting high solubility and very weak affinity with cyclodextrins.

In our efforts to design an experimental strategy to improve accuracy, we also evaluated the contribution of each protocol when coupled to the titration-release approach. Nonetheless, no combination of three different experiments seems to systematically and significantly decrease the uncertainty, if compared to combinations of titration and release experiments. In terms of accuracy, it seems that the titration-release approach should be considered as the gold standard. However, in terms of reliability, implementation of each non-conventional experiment for a given complex may provide additional information on the adequacy between theoretical model and experimental reality. This may be especially helpful if another chemical equilibrium is suspected to occur.

### Competitive experiments

The second part of this work is devoted to the study of inclusion compounds in competitive conditions. In these experiments, a given complex is characterized in the presence of a second host or of a second guest. Before the interpretation of such a competitive experiment involving the three partners, it is necessary to realize the individual study of the second complex by a titration procedure or preferably by a titration-release approach. It has to be mentioned that ITC competitive protocols have been initially developed for the characterization of very strong complexes [12]; in this case, the sigmoidal shape of the isotherm becomes too linear to allow an accurate analysis of each parameter. As such analytical problem occurs for binding constant superior to 10^7^–10^8^ M^−1^, competitive methods, to the best of our knowledge, have not been applied to cyclodextrin complexes, since their stability is generally inferior to 10^5^ M^−1^.

**Figure 4:**
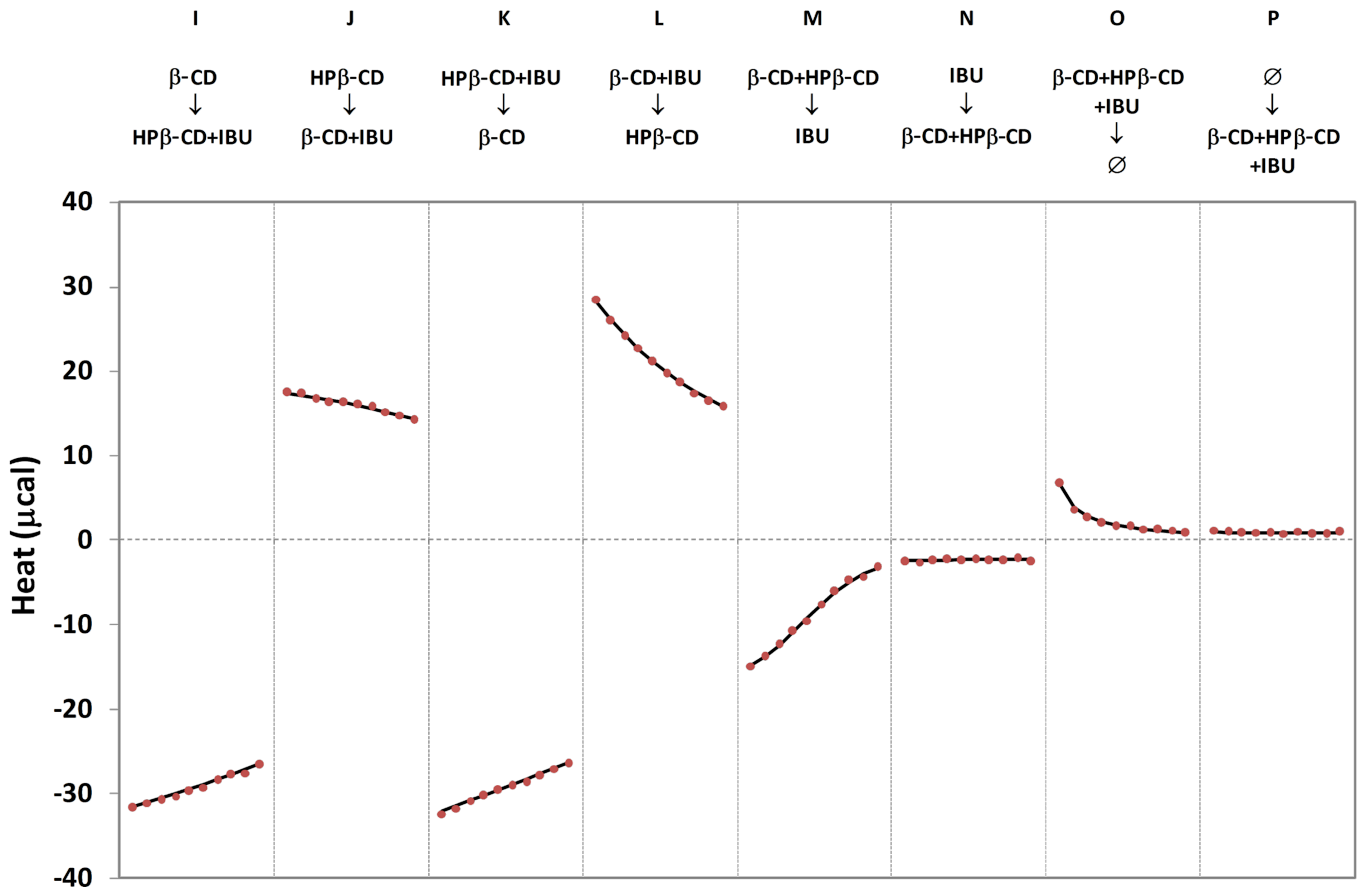
Competitive ITC isotherms for the HPβ-CD-β-CD-IBU system at 283 K. Each protocol (from I to P) is defined by the respective content of syringe (top) and cell (bottom). Ø indicates the buffer solution. Lines and dots correspond to theoretical and experimental data, respectively. The x axis corresponds to the injection number (10 injections per isotherm).

In order to evaluate the potential efficiency of competitive experiments in the field of cyclodextrin complexes, we estimated the corresponding uncertainty in the case of hypothetical complexes for various concentrations of the three partners and for various values of formation constants. In the case of complexes which are well characterized by the titration-release approach, none of our trials have shown any significant improvement by a competitive strategy, for both host and guest competitors. This result reinforces the initial hypothesis that competition should be restricted to problematic studies. However, there are some circumstances under which the direct ITC protocols fail to characterize cyclodextrin complexes, and then competitive methods may be of valuable aid. We observed this kind of behavior when the studied inclusion complex is nearly athermic (inclusion enthalpy close to 0 cal·mol^−1^). For example, this is the case for the HPβ-CD-IBU complex at 283 K: under these conditions, titration and release signals are not significantly different from blank experiments. As a consequence, the uncertainty on the thermodynamic parameters is infinite, and such a complex cannot be studied by ITC. On the other hand, the β-CD-IBU complex is easily characterized at 283 K, and, as a result, it could act as a competitive system. Within this scope, we evaluated the potential of the HPβ-CD-β-CD-IBU system to characterize the HPβ-CD-IBU complex. For the design of competitive protocols, each ITC compartment may contain none, one, two or three of the HPβ-CD, β-CD and IBU partners. When we consider all possible combinations, eight competitive experiments may be investigated, if we exclude the less promising experiments involving the simultaneous presence of a given partner in the two compartments. These eight experiments are illustrated in Figure 4. 5 mM solutions were used for cyclodextrins, while concentrations equal to 0.5 mM and 4.6 mM were employed for IBU in the absence of and in the presence of cyclodextrins, respectively. Again, the unified data treatment managed to describe all competitive experiments, in addition to its previously demonstrated ability to fit non-competitive data. Global analysis leads to an inclusion enthalpy Δ*H*_HPβ-CD-IBU_ which is not significantly different from 0 (−3 ± 12 cal·mol^−1^), thus confirming the athermic character of this complex. The formation constant *K*_HPβ-CD-IBU_ is evaluated to 5477 M^−1^, again with a high accuracy (±146 M^−1^). This stability uncertainty rises to ±426 M^−1^ if we take into account an uncertainty of 5% on each total concentration. Such results are particularly remarkable for an athermic complex, furthermore with a low Wiseman criterion (close to 3).

Among the eight combinations, four protocols induce the transfer of IBU from one cyclodextrin to the other, the initial complex being formed in the cell (protocol I and J, called “competitive titration”) or in the syringe (protocol K and L, called “competitive release”). In these cases, exothermic peaks are obtained when IBU is transferred from the HPβ-CD to the β-CD cavity (protocol I and K), since the variation of inclusion enthalpy (ΔΔ*H* = Δ*H*_β-CD-IBU_ -Δ*H*_HPβ-CD-IBU_) is negative. In the reverse case, i.e., protocols J and L, endothermic peaks are observed. Two other protocols involve simultaneous complexation of IBU by the two cyclodextrins (protocols M and N, called “double titration” and “double reverse titration”, respectively), thus leading to negative peaks. Finally, two protocols use the buffer solution to dilute a mixture of the three partners (protocols O and P, called “double release” and “double dilution”, respectively), which induces the simultaneous dissociation of both complexes, resulting in positive peaks. The eight experiments lead to very dissimilar isotherms, which can be modulated furthermore by varying the relative total concentrations of the various partners. Under the conditions used in this study, the stability of the HPβ-CD-IBU complex may also be obtained with high accuracy by individual analysis for each experiment (Table 2). While the uncertainty is infinite for non-competitive protocols, a dramatic fall is observed when using the competitive system with uncertainty on *K* inferior to 20% for protocols I to M. As the accuracy of competitive experiments is depending on multiple factors (binding constants, inclusions enthalpies, concentrations), it has to be pointed out that each inclusion compound would require such theoretical investigations in order to design the best competitive protocol.

**Table 2:** Theoretical uncertainties of *K* for an individual analysis of the eight competitive ITC protocols described in Figure 4, in the case of the HPβ-CD-IBU complex at 283 K (total concentrations are considered as perfectly known).

Protocol	I	J	K	L	M	N	O	P

*t*·δ*K*	16%	15%	10%	6%	9%	>100%	88%	>100%

This study on the HPβ-CD-IBU system at 283 K demonstrates that some inclusion compounds which cannot be studied by direct methods may be accurately characterized by competitive protocols. Such an improvement may also be exploited, for instance, if a weak solubility is observed for both host and guest. In this case, no classical titration may be realized, since none of the individual concentrations is sufficient to be used in the syringe. To overcome such limitation, it is possible to solubilize the guest by the use of a soluble cyclodextrin and to inject the resulting solution on the poorly soluble cyclodextrin placed inside the cell. The feasibility of this experiment is illustrated in Figure 4 by protocol L, which can be efficiently undertaken for various concentrations of cyclodextrin in the cell. In the case of the HPβ-CD-β-CD-IBU system, an uncertainty of 27% is obtained for *K*_HPβ-CD-IBU_ when using a HPβ-CD concentration equal to 0.5 mM. Considering both this rather weak cyclodextrin concentration and the athermic character of this complex, such accuracy is at the same time unexpected and very satisfying.

At last, the HPβ-CD-IBU system was studied over the 283–313 K range, with a 7.5 K temperature step, both by the titration-release and competitive approaches. The competitive approach have been realized on the basis of protocol L (injections of a β-CD-IBU mixture on a HPβ-CD-solution), the β-CD-IBU complex being characterized by titration-release experiments. The resulting thermodynamic data are shown in Table 3.

**Table 3:** Thermodynamic data for the HPβ-CD-IBU complex obtained by a global analysis of the competitive approach and the titration-release approach (considering a 5% uncertainty on each total concentration). Data for the β-CD-IBU complex correspond to the global analysis of titration-release experiments.

	HPβ-CD-IBU				β-CD-IBU	
	Competitive approach		Titration-release approach		Titration-release approach	
	*K* ± *t*·δ*K* (M^−1^)	Δ*H* ± *t*·δΔ*H* (cal·mol^−1^)	*K* ± *t*·δ*K* (M^−1^)	Δ*H* ± *t*·δΔ*H* (cal·mol^−1^)	*K* ± *t*·δ*K* (M^−1^)	Δ*H* ± *t*·δΔ*H* (cal·mol^−1^)

283.0 K	5457 ± 648	−9 ± 21	5566 ± 252	−2 ± 15	11985 ± 595	−1954 ± 64
290.5 K	5360 ± 637	−786 ± 32	5452 ± 248	−921 ± 33	10804 ± 536	−2576 ± 84
298.0 K	5076 ± 603	−1733 ± 65	5146 ± 234	−1750 ± 59	9502 ± 471	−3329 ± 110
305.5 K	4637 ± 551	−2649 ± 100	4713 ± 215	−2502 ± 85	8153 ± 405	−4069 ± 137
313.0 K	4118 ± 489	−3387 ± 130	4188 ± 191	−3505 ± 120	6855 ± 340	−4726 ± 163
Δ*C_p_*	−114 cal·mol^−1^·K^−1^		−115 cal·mol^−1^·K^−1^		−94 cal·mol^−1^·K^−1^	

As might be expected from the negative inclusion enthalpy of the β-CD-IBU complex at 283 K, the affinity of β-CD to IBU decreases with temperature. The HPβ-CD-IBU complex follows the same trend, as its inclusion enthalpy, which is negligible at 283 K, becomes negative for temperatures superior to 283 K. The decreasing enthalpy variation observed for both complexes results from negative values of heat capacity variations (see Table 3), which are generally associated with the occurrence of hydrophobic forces [33]. On the methodological point of view, it has to be pointed out that the titration-release approach becomes applicable for temperatures above 283 K, since the HPβ-CD-IBU inclusion enthalpy is not negligible anymore. Therefore, the HPβ-CD-IBU thermodynamic parameters may even be evaluated at 283 K by means of the non-competitive strategy by means of a global analysis of the titration-release results obtained for HPβ-CD-IBU at all temperatures. As can be seen in Table 3, the accuracies resulting from the different approaches are very satisfying, especially when considering that inclusion enthalpies of the HPβ-CD-IBU complex are rather low and even negligible at 283 K. Finally, titration-release and competitive strategies lead to highly consistent results on *K*, Δ*H* and Δ*C_p_*, confirming the strong reliability of both approaches and demonstrating the complementarity of the various non-conventional protocols.

## Conclusion

If the titration protocol constitutes the overwhelming majority of the published ITC studies on cyclodextrin complexes, our work clearly demonstrates that non-conventional experiments can greatly improve the calorimetric characterization. In particular, the release protocol has been found to be more accurate than titration for a wide range of cyclodextrin inclusion compounds. More strikingly, the global analysis of coupled release and titration experiments should be considered as the new standard approach, as it is systematically more efficient than titration. In the specific case of guests with high solubility and low affinity for cyclodextrins, thermodynamic characterization may be improved by the coupling of non-conventional experiments, such as reverse titration and annihilated release. In addition, ITC competitive experiments, which are totally absent from the cyclodextrin literature, may be very useful when the non-competitive experiments fail to characterize a given complex, for instance in the case of weak inclusion enthalpy or weak host solubility. Furthermore, the use of various ITC experiments to study an inclusion compound should be considered as a helpful tool to ensure that the theoretical model really corresponds to the observed equilibrium. We believe that the strategies presented in this work should help to improve the accuracy and reliability of thermodynamic data, not only for easily accessible complexes but also when ITC studies approximate their limits of detection.

## Experimental

### Materials

Ibuprofen (racemic mixture, sodium salt), sodium hydroxide and potassium dihydrogen phosphate were purchased from Acros Organics. β-CD, HPβ-CD (with a mean of 5.6 substitutions per cyclodextrin) were purchased from Wacker Chemie (Lyon, France). The water content (10% for β-CD, 6% for HPβ-CD) has been taken into account when preparing solutions. All products were of analytical grade and used as received. Distilled deionized water was used throughout this work.

### ITC experiments

An isothermal calorimeter (ITC_200_, MicroCal Inc., USA) was used for simultaneously determining the formation constant and the inclusion enthalpy of the studied complexes at five temperatures (283, 290.5, 298, 305.5 and 313 K). Degassed solutions (phosphate buffer, pH 6.5) were used in both cell (202.8 μL) and syringe (40 μL). For all protocols, experiments were implemented as follows. After the addition of an initial aliquot of 1 μL, 10 aliquots of 3.7 μL of the syringe solution were delivered (over 7.4 s for each injection). The time interval between two consecutive injections was 150 s and the agitation speed was 1000 rpm for all experiments. The resulting heat flow was recorded as a function of time. In addition, the heat of dilution (for each partner) was eliminated by subtracting the raw signal obtained for the corresponding blank titrations (i.e., only one partner in cell or syringe, the other compartment being filled with buffer). The peak area following each addition was obtained by integration of the resulting signal and was expressed as the heat effect per injection. Binding constants and inclusion enthalpies were finally determined by nonlinear regression analysis of the binding isotherms by means of a dedicated homemade program (Excel spreadsheet). All conventional and none conventional experiments were realized by attributing, to the cell and syringe, one of the 8 prepared solutions (buffer, IBU, β-CD, HPβ-CD, IBU + β-CD, IBU + HPβ-CD, βCD + HPβ-CD, IBU + β-CD + HPβ-CD). Effective concentrations are presented in the main text.

### Data treatment

The data treatment consists of the postulation of values for formation constants and inclusion enthalpies, the simulation of the heat obtained for each injection, and the minimization of the difference between experimental and simulated heats by varying the initial thermodynamic parameters.

If we consider two molecules (for instance two host molecules: β-CD and HPβ-CD) which compete for inclusion with a third molecule (for instance one guest molecule: IBU), equilibria, formation constants and total concentrations are given by Equations 2–8 :

[2]
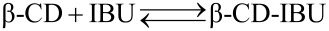


[3]
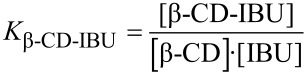


[4]
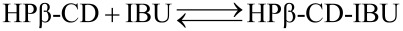


[5]
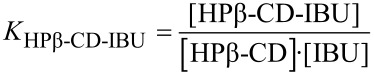


[6]
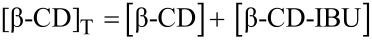


[7]
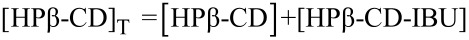


[8]
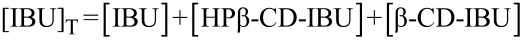


These relations are effective for both cell and syringe. In addition, the titrand total concentration may be multiplied by a corrective factor (*n*), equal to 1 by default, in order to obtain the effective concentration by the use of the fitting procedure. According to the low values of the Wiseman criterion for cyclodextrin complexes, this *n* parameter is kept fixed for the evaluation of the uncertainty in binding constant and inclusion enthalpy.

While the syringe content remains unchanged during the experiment, the cell content is dependent on the injections. The total concentrations for each partner (denoted as [X]_T_) within the cell may be described at any time (injection i) of the experiment by Equation 9 [33] :

[9]
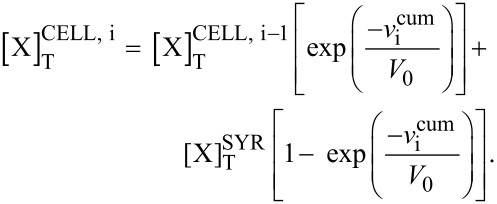


The superscripts CELL and SYR indicate quantities relative to the cell and the syringe, respectively. *V*_0_ and *v*_i_^cum^ corresponds to the cell volume and to the cumulated injected volume for injection i, respectively.

On the basis of the total concentrations, the concentrations of the inclusion compounds may be calculated according to Wang [34], by using Equations 10 and 11, which may be independently applied, to both cell and syringe:

[10]
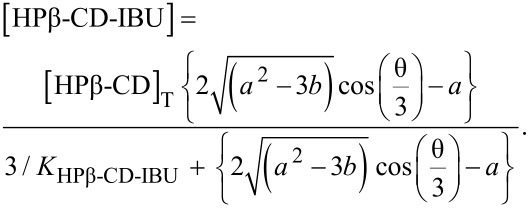


[11]
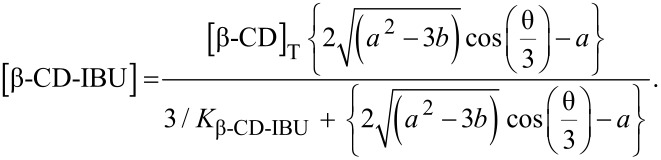


With :

[12]
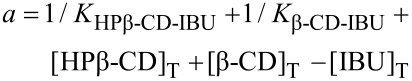


[13]
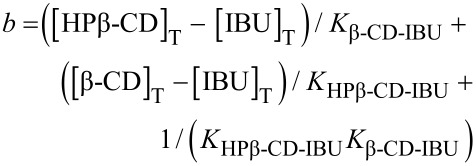


[14]
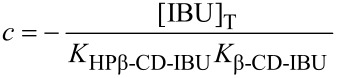


[15]
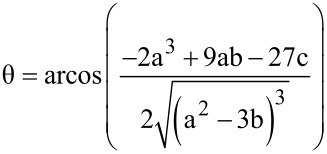


The knowledge of these concentrations in the syringe and in the cell (before and after each injection) allows the calculation of the associated heat, on the basis of Equation 16:

[16]
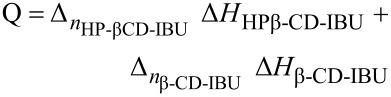


with Δ*n* corresponding to the variation of the number of complexes in the cell. If we combine the equations developed by Tellinghuisen [35] for titration and Heerklotz [14] for release, Δn may be described by Equation 17, for each complex:

[17]
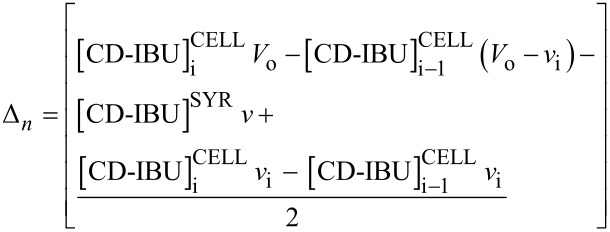


Equations 16 and 17 hold true independent from the solutions which are employed in the cell and the syringe, for both competitive and non-competitive conditions. Indeed, the absence of one of the hosts brings the previous relationships to the simple case of a unique 1:1 complex. Equation 16 corresponds to the regression function for the least square fitting of the binding isotherms. This fitting is realized by means of the Newton–Raphson algorithm, which minimizes the residual sum of squared deviation between experimental and theoretical data by varying association constants and inclusion enthalpies.

### Global analysis

Data treatment may be implemented for one or several experiments with one set of thermodynamic parameters. If more than one experiment is analyzed, the least square fitting is simultaneously applied to all isotherms. If the various experiments are investigated at the same temperature, the global analysis relies, for each complex, on one formation constant (*K*) and one inclusion enthalpy (Δ*H*). If several experiments are carried out at different temperatures (*T*_i_), the binding enthalpies (Δ*H*^Ti^) are optimized for each temperature, while the affinity is fitted on the basis of only one formation constant at a reference temperature *T*_0_ (*K*^T0^). The association constants for the other temperatures are then calculated by trapezoidal numerical integration of the van’t Hoff expression according to Freiburger [36]:

[18]
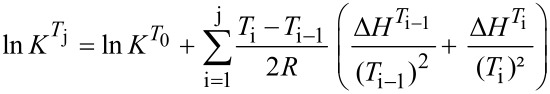


When all binding enthalpies are known, the heat capacity variation may be estimated by linear least square according to Equation 19:

[19]
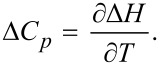


### Simulation of accuracy

Uncertainties on the thermodynamic parameters are determined by using the a priori variance–covariance matrix *V* [37]. *V* presumes prior knowledge about the random error structure of the data; it is computed on the basis of exact fitting data for the model in question. The diagonal elements in *V* correspond to the variances of the optimized binding parameters. *V* is given by:

[20]
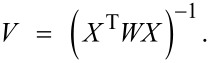


The elements of the matrix *X* are *X*_ij_ = (∂*F*_i_/∂β_j_), where *F* expresses the fit function and β the adjustable parameters. The partial derivatives are evaluated at each value of the independent variable by using the final (iterated) values of the fit parameters.

The weight matrix *W* is diagonal, with a dimensionality equal to the number of data points and elements which are the inverse data variances, *W*_ii_ = σ_i_^−2^. The typical standard deviation of heat (σ) was obtained from independent repeatability studies and was estimated to be 0.2 μcal for our ITC_200_ calorimeter.

The uncertainties of the binding constants and inclusion enthalpies are expressed as the standard deviations (δ*K* and δΔ*H*, respectively) multiplied by the student *t* factor (1.96).

Simulations of the accuracy were carried out for both the real systems studied in this work and hypothetical systems. In the latter case, corresponding binding isotherms were simulated by the postulation of thermodynamic parameters and experimental conditions and by calculating the heat according to the data treatment section.

In the case of a global analysis, all isotherms are used simultaneously in the a priori variance–covariance matrix. As a result, accuracy benefits from information over all experiments. In the case of competitive experiments, the accuracy on the studied complex takes into account the accuracy on the competitive complex (and reciprocally).

## Supporting Information

File 1 Complementary data of uncertainty on *K* and Δ*H* for various protocols.
