# Supplementary material for: Improving ITC studies of cyclodextrin inclusion compounds by global analysis of conventional and non-conventional experiments
Source: Beilstein J Org Chem. 2014 Nov 11;10:2630–41. doi: 10.3762/bjoc.10.275 (PMC4273236; doi:10.3762/bjoc.10.275)
Supplement: File 1 — Complementary data of uncertainty on K and ΔH for various protocols. [file Beilstein_J_Org_Chem-10-2630-s001.pdf]

Supporting Information

for

Improving ITC studies of cyclodextrin inclusion compounds by global analysis of conventional and non-conventional experiments

Eléonore Bertaut[1,2], David Landy[1,2] *

Address: [1] Univ Lille Nord de France, F-59000 Lille, France and [2] ULCO, UCEIV, F-59140 Dunkerque, France

Email: David Landy - landy@univ-littoral.fr

* Corresponding author

Complementary data of uncertainty on *K* and Δ*H* for various protocols

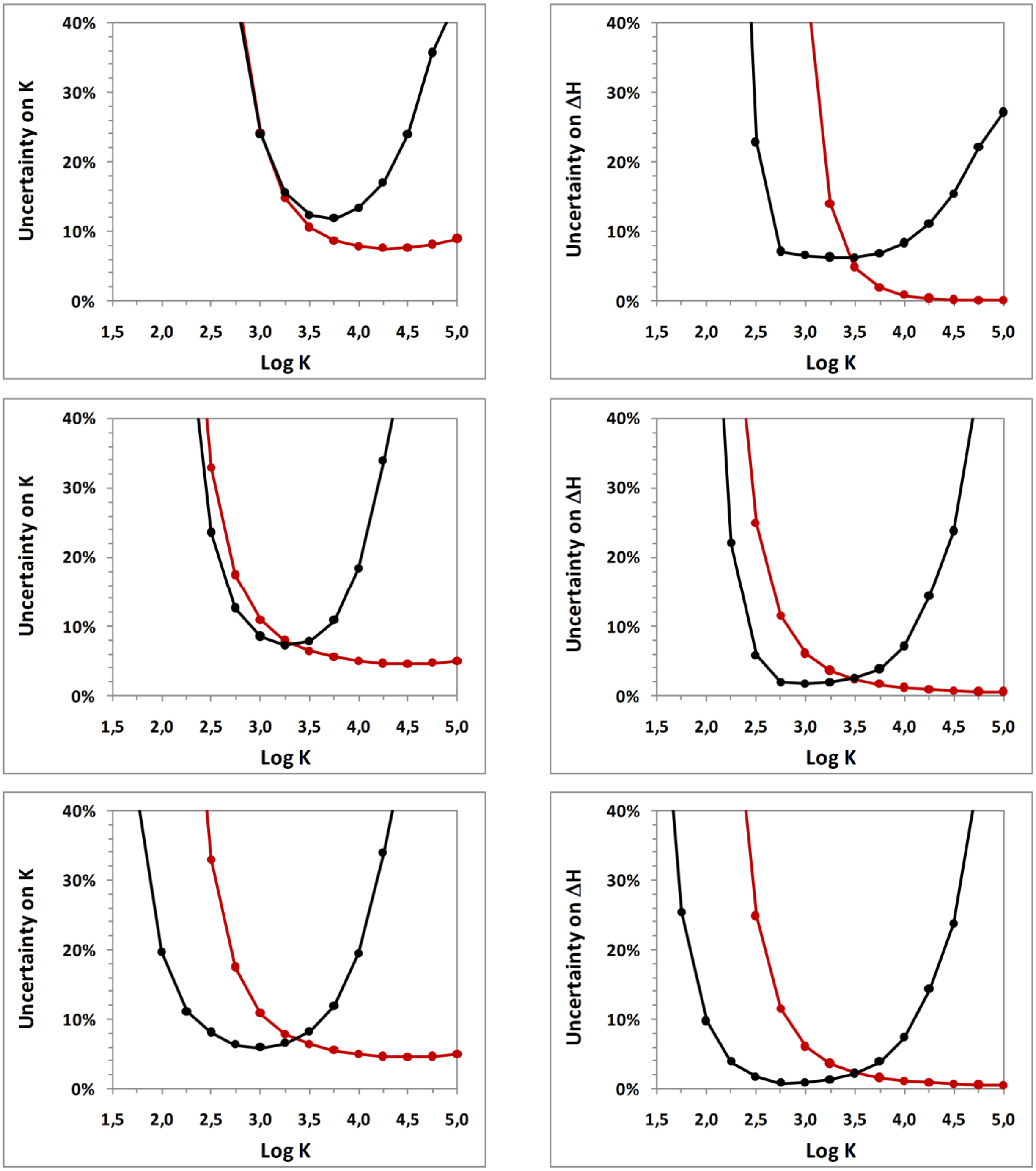

Figure S1: Theoretical uncertainty on K and ΔH as a function of log K for titration (red curve) and release (black curve) protocols, for aqueous guest solubility equal to 0.1 (top), 0.5 (middle) and 5 mM (bottom) and for an inclusion enthalpy equal to −3000 cal·mol^{-1} (Total concentrations considered as perfectly known).

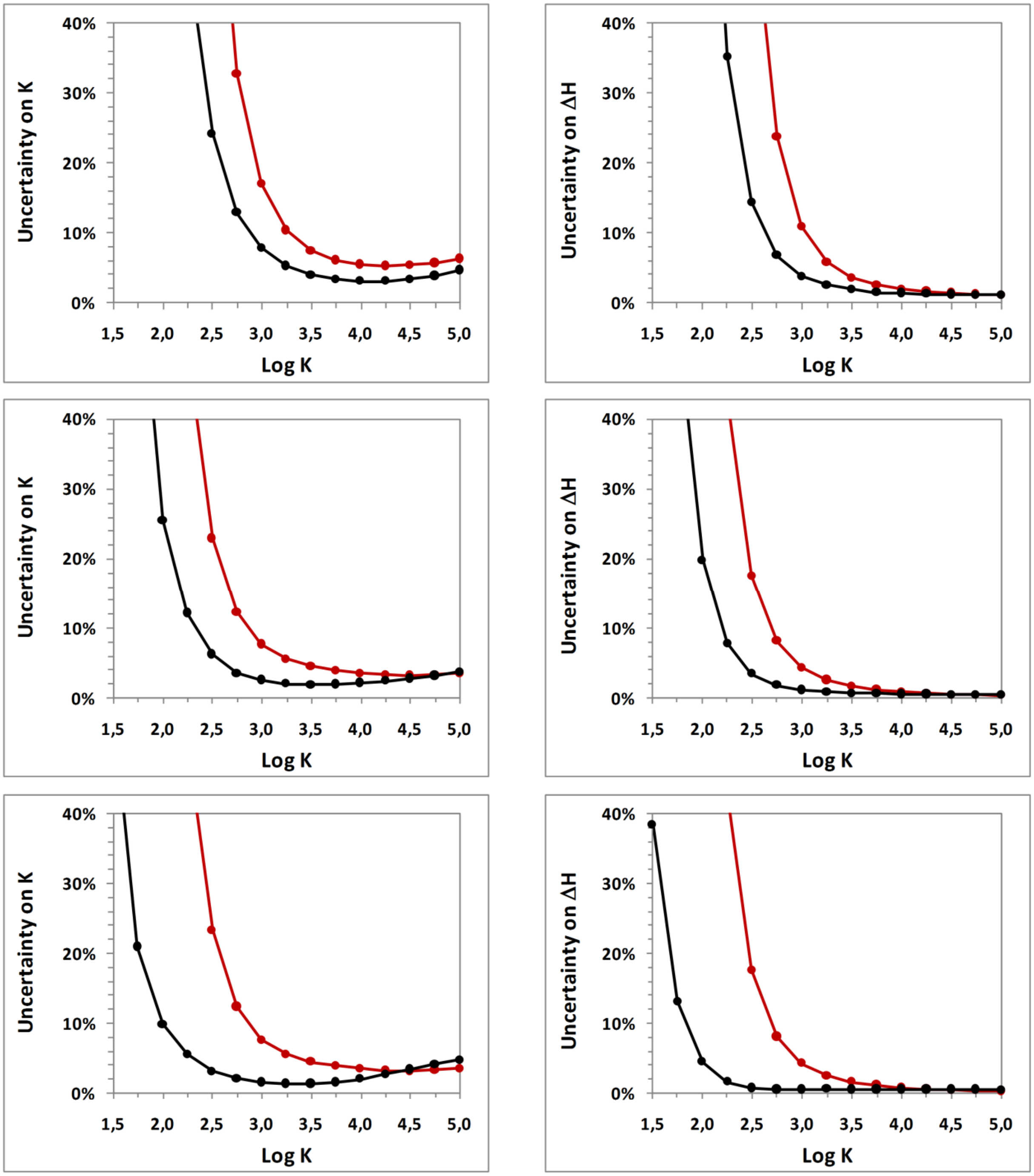

Figure S2: Theoretical uncertainty on K and ΔH as a function of log K for titration-titration (red curve) and titration-release (black curve) approaches, for an aqueous guest solubility equal to 0.1 (top), 0.5 (middle) and 5 mM (bottom) and for an inclusion enthalpy equal to −3000 cal·mol^{-1} (Total concentrations considered as perfectly known).

Table S1: Theoretical uncertainties on *K* for titration-titration, titration-release and reverse titration-annihilated release approaches (aqueous guest solubility equal to 2 mM, inclusion enthalpy equal to −3000 cal·mol^{-1}). Total concentrations considered as perfectly known.

		Titration-titration		Titration-release		reverse titration-annihilated release	
Log K	K (M^{-1})	t*δK	t*δΔH	t*δK	t*δΔH	t*δK	t*δΔH
1,50	32	>100%	>100%	93%	75%	37%	34%
1,75	56	>100%	>100%	38%	26%	17%	13%
2,00	100	>100%	>100%	16%	9%	8%	6%
2,25	178	51%	43%	8%	3%	4%	3%
2,50	316	23%	18%	4%	1%	3%	2%
2,75	562	12%	8%	2%	1%	3%	1%
3,00	1000	8%	4%	2%	1%	3%	1%
3,25	1778	6%	3%	2%	1%	4%	1%
3,50	3162	4%	2%	2%	1%	4%	1%
3,75	5623	4%	1%	2%	1%	5%	1%
4,00	10000	3%	1%	2%	1%	6%	1%
4,25	17783	3%	1%	2%	1%	7%	1%
4,50	31623	3%	1%	3%	1%	8%	1%
4,75	56234	3%	1%	3%	1%	10%	1%
5,00	100000	3%	1%	4%	1%	12%	1%
